# Divergent Mechanisms for PPARγ Agonism in Ameliorating Aging-Related Versus Cranial Irradiation-Induced Context Discrimination Deficits

**DOI:** 10.3389/fnagi.2019.00038

**Published:** 2019-03-14

**Authors:** Ibdanelo Cortez, Larry Denner, Kelly T. Dineley

**Affiliations:** ^1^Department of Neurology, The University of Texas Medical Branch at Galveston, Galveston, TX, United States; ^2^Department of Internal Medicine, The University of Texas Medical Branch at Galveston, Galveston, TX, United States

**Keywords:** PPAR gamma, hippocampus, context discrimination, adult neurogenesis, microglia, BrdU

## Abstract

A major aspect of mammalian aging is the decline in functional competence of many self-renewing cell types, including adult-born neuronal precursors in a process termed neurogenesis. Adult neurogenesis is limited to specific brain regions in the mammalian brain, such as the subgranular zone (SGZ) of the hippocampus. Alterations in adult neurogenesis appear to be a common hallmark in different neurodegenerative diseases including Alzheimer’s disease (AD). We and others have shown that PPARγ agonism improves cognition in preclinical models of AD as well as in several pilot clinical trials. Context discrimination is recognized as a cognitive task supported by proliferation and differentiation of adult-born neurons in the dentate gyrus of the hippocampus that we and others have previously shown declines with age. We therefore postulated that PPARγ agonism would positively impact context discrimination in middle-aged mice via mechanisms that influence proliferation and differentiation of adult-born neurons arising from the SGZ. To achieve our objective, 8-months old mice were left untreated or treated with the FDA-approved PPARγ agonist, rosiglitazone then tested for context discrimination learning and memory, followed by immunofluorescence evaluation of hippocampal SGZ cell proliferation and neuron survival. We found that PPARγ agonism enhanced context discrimination performance in middle-aged mice concomitant with stimulated SGZ cell proliferation, but not new neuron survival. Focal cranial irradiation that destroys neurogenesis severely compromised context discrimination in middle-aged mice yet rosiglitazone treatment significantly improved cognitive performance through an anti-inflammatory mechanism and resurrection of the neurogenic niche. Thus, we have evidence for divergent mechanisms by which PPARγ agonism impinges upon aging-related versus cranial irradiation-induced deficits in context discrimination learning and memory.

## Introduction

The Peroxisome Proliferator-activated Receptor gamma (PPARγ) is a ligand-modulated nuclear transcription factor and a therapeutic target for treating insulin resistance in type-2 diabetes patients. As such, FDA-approved drugs that activate PPARγ are highly effective for treating type-2 diabetes in the form of the thiazolidinediones (TZDs) pioglitazone (Actos^TM^) and rosiglitazone (Avandia^TM^; GlaxoSmithKline plc., Brentford, United Kingdom). While much is known regarding the role of PPARγ in peripheral tissues, e.g., adipocytes and skeletal muscle (Desvergne and Wahli, 1999; Wolf, 2004), its role in brain function is expanding. PPARγ is primarily expressed in neurons and somewhat in astrocytes; PPARγ is essentially absent from microglia (Warden et al., 2016). This strong neuronal signature raises the possibility that PPARγ agonism predominantly targets neurons to alleviate aging-related neurodegenerative processes.

We and others have demonstrated that PPARγ agonism improves hippocampus-dependent cognitive performance in Alzheimer’s disease (AD) mouse models, predominantly in tasks that require intact hippocampal ERK signaling (Pedersen et al., 2006; Escribano et al., 2009; Rodriguez-Rivera et al., 2011; O’Reilly and Lynch, 2012). Cognitive enhancement has been shown to be accompanied by improved AD biomarker profiles: alleviation of amyloid and tau pathology (Escribano et al., 2010; Jiang et al., 2012; Mandrekar-Colucci et al., 2012; O’Reilly and Lynch, 2012; Kummer et al., 2014), reduced neuroinflammation (Escribano et al., 2010; Yin et al., 2013; Prakash and Kumar, 2014), increased antioxidant protection (Nicolakakis et al., 2008; Yin et al., 2013), amelioration of central insulin resistance (Masciopinto et al., 2012; Yin et al., 2013), and normalization of several gene transcripts and proteins related to plasticity in the hippocampus, including reversal of down-regulated PPARγ (Denner et al., 2012).

In this study, we set out to establish whether PPARγ agonism improved age-dependent decline in context discrimination (Frankland, 1998; Sahay et al., 2011a; Cortez et al., 2017), a cognitive task supported by proliferation and differentiation of adult-born neurons in the dentate gyrus of the hippocampus (Aimone et al., 2010; Wu et al., 2015; Cortez et al., 2017).

To achieve our objective, 8-months old B6SJL mice were left untreated or treated with the FDA-approved PPARγ agonist, rosiglitazone (RSG) followed by context discrimination testing at 9-months of age and *post hoc* immunofluorescence evaluation of hippocampal subgranular cell proliferation and neuron survival 45 days post-injection (dpi) with bromodeoxy-uridine (BrdU). We found that while context discrimination was enhanced in RSG-treated mice and that RSG stimulated hippocampal progenitor cell production, treatment did not promote neuronal differentiation or survival from these progenitors. Focal cranial irradiation was then employed to destroy adult hippocampal neurogenesis. PPARγ treatment under these conditions enhanced context discrimination performance and reduced Iba-1-positive microglia in the hippocampus. These findings indicate divergent mechanisms for PPARγ agonism in ameliorating aging-related vs. cranial irradiation-induced deficits in context discrimination learning and memory.

## Materials and Methods

### Animals

B6SJL mice were bred and aged in the Animal Resource Center at the University of Texas Medical Branch at (UTMB). UTMB Animal Resource Center Facilities operate in compliance with the USDA Animal Welfare Act, the Guide for the Care and Use of Laboratory Animals, under OLAW accreditation, and IACUC approved protocols. Mice were separated by sex, identified upon weaning, and allowed food and water *ad libitum*. The animal colony was maintained at ambient temperature (21–25°C) and humidity (45–50%) on a 12 h light–dark cycle (lights on 0700–1900 h); mice were housed *n* ≤ 5/cage. Animals were randomly assigned to control (male = 7, female = 10) RSG treatment (male = 8, female = 9), radiation treatment (male = 6, female = 7) and RSG-radiation treatment (male = 9, female = 7) groups. All testing was conducted during the light cycle. Context fear discrimination was performed on two separate cohorts of randomly assigned animals. No animals were omitted from analysis based upon behavioral performance.

Body weights (g) taken immediately before behavioral analysis were not different among groups in this study; Control (mean = 30.94 g), RSG (mean = 30.7 g), Radiation (mean = 30.31 g), Radiation+RSG (mean = 31.75 g); (*F*_(1,59)_ = 0.49, *p* = 0.48).

### PPARγ Agonist Treatment

Male and female B6SJL mice were treated with RSG as previously described (Rodriguez-Rivera et al., 2011). RSG was milled in standard rodent chow (Bio-Serv) at 30 mg/kg chow, a dose chosen based on previous studies validated for food intake, body weight, and safety (Pedersen et al., 2006; Rodriguez-Rivera et al., 2011; Denner et al., 2012; Jahrling et al., 2014; Nenov et al., 2014, 2015).

### BrdU Injection

**FIGURE 1:**
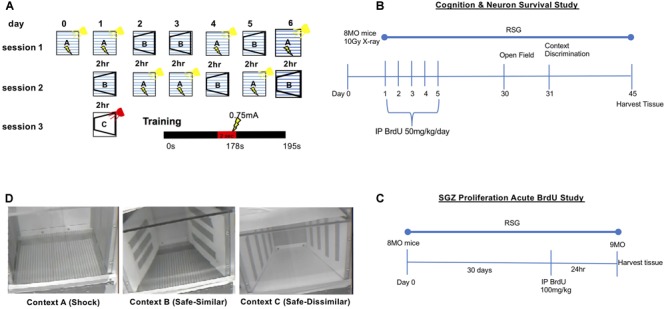
Context discrimination fear conditioning paradigm. **(A)** Schematic illustrating context presentations across 6 days. On training day 0, subjects were placed in shock context A and allowed to explore the shock context for 178 s at which point a 2 s 0.75 mA foot shock was delivered; subjects are kept in context A for an additional 15 s, totaling 195 s training. On test days 1–6, subjects were randomly presented with either context A or B. In context A, mice receive the footshock at the 178 s mark of the 195 s session. In context B, mice are allowed to explore for 195 s. Between context presentations, a 2-h inter-trial interval was inserted during which time mice are returned to their home cage. As a control for baseline propensity to freeze, on test day 1 mice are presented with novel context, C. Total percent freezing was measured for each mouse in every context (lightning bolts symbolize foot shock delivery). **(B)** Timeline for the cognition and neuron survival study. Briefly, 8 MO animals were IP injected with 50 mg/kg BrdU 24 h after irradiation, once a day for 5 consecutive days. Behavioral testing commenced on day 30. Tissue harvest was performed on day 45. **(C)** For the SGZ proliferation acute BrdU study, mice that *did not* receive focal cranial irradiation were also left untreated or treated with RSG beginning at 8 MO. These animals were injected with a single dose of BrdU (100 mg/kg) 24 h before tissue harvest. **(D)** Digital images of Contexts A, B, and C used in for context discrimination as described in panel **A**. Panel **(A)** adapted and reprinted from Cortez et al. (2017), copyright 2016, with permission from Elsevier.

All 8 MO B6SJL (behavioral cohort) mice were given a total of 5 intraperitoneal injections (IP) of the thymidine analog BrdU, 50 mg/kg/day (Figure 1B). Tissue was harvested 45 days after final injection to study survival of new born neurons. In a separate cohort (5 mice/group), an acute BrdU analysis was accomplished by treating 9 MO animals in control and RSG (45 days w/RSG) groups with a single IP injection of BrdU at 100 mg/kg and tissue harvested 24 h later (Figure 1D).

### Focal Cranial Irradiation

Isofluorane-anesthetized 8-month old mice were exposed to a single 10 Gy X-ray dose at ∼250 cGy/min (or sham) directed at the cranium (0, –2.5 bregma). Mice were either left untreated or treated with RSG and context discrimination testing 45 days later.

### Behavioral Tests

#### Open Field

The open field test was used to measure natural exploration and ambulation in this study 30 days post-irradiation treatment at 9 months of age. Mice were placed in the center of a (L × W × H) (38 × 38 × 38 cm) white Plexiglas^TM^ box and allowed to explore for 10 min. Digital video-based data capture and analysis was achieved with TopScan software (Clever Sys) using central (12.6 cm^2^) and peripheral (25.4 cm^2^) arenas to track exploratory behavior. Similar methods were used in previous reports from our lab (Hernandez et al., 2014; Cortez et al., 2017). Briefly, the software was programmed to track speed, total distance, and time spent in center and peripheral arenas for each animal. After each trial, mice were placed back in their home cage and open field boxes were cleaned with 70% ethanol before the next animal was tested.

#### Context Discrimination

Subsequently, all groups were tested in the context fear discrimination paradigm adapted from Frankland (1998) and Sahay et al. (2011a). This cognitive task measures an animal’s ability to distinguish between two similar yet different environmental contexts (Frankland et al., 1998; Figure 1) On training day 0 each subject was placed in a standard mouse fear conditioning chamber (Med Associates) which served as context A, the shock context. Every time the mouse experienced context A (aversive context) training/testing ensued for 195 s during which time a single 2 s 0.75 mA shock was delivered through an electrified grid floor at the 178 s mark; mice then had an additional 15 s to explore the context. Mice were returned to their home cages upon termination of the task.

For testing days 1–6, mice were randomly assigned to first experience either context A, or a similar yet non-identical “safe” context, context B. Context B (safe context) resembled context A in terms of the grid floor being exposed. Context B contained cardboard inserts, vanilla extract (2 μL pipetted onto a KimWipe^®^), and the chamber light and fan were turned off. Like context A, mice were placed in context B for 195 s, however, no shock was delivered. A 2 h inter-trial interval between each context was utilized for each subject for each day of testing. Following each session, 70% ethanol was used to thoroughly wipe down Context A and 95% isopropyl alcohol was used to clean Context B.

Digital video-based data capture and analysis with FreezeFrame software (Actimetrics) was used to assess freezing behavior. Freezing was determined by setting motion index above background motion and motion detected from animals natural breathing. Once session was complete, data was exported to an excel file and average % freezing was used in subsequent analysis. Discrimination ratios (% freezing in Context B ÷ % freezing in Context A+B) were calculated for each group on each test day.

Analysis of total %freezing during the training session on day 0 and in the novel Context C on day 1 served as controls for fear generalization. Control Context C consisted of a novel context in which several features were changed from Context A and B, most important being concealment of the metal grid floor that delivered the foot-shock with a white plexiglass cover.

### Tissue Collection

Once behavioral testing was complete, mice were anesthetized with ketamine/xylazine and transcardially perfused with 0.1 M Phosphate Buffer Saline (1× PBS) for 3 min and then with the fixative solution 4% paraformaldehyde in 1× PBS for another 5 min. Animals were decapitated, brains harvested, then post-fixed in fixation solution and incubated at 4°C. After overnight post-fixation, brains were submerged in 30% sucrose and stored for immunofluorescence evaluation of BrdU uptake and incorporation, new neuron production, neuron survival, and microglia response. Subjects (5 mice/group) used in Ki67, doublecortin and new born neuron survival immunostaining analysis were randomly selected and sex distribution were: control group (male = 2, female = 3), RSG group (male = 2, female = 3), irradiation group (male = 2, female = 3), and RSG-irradiation group (male = 3, female = 2). Subjects in proliferation acute BrdU staining were: control group (male = 1, female = 4), RSG group (male = 4, female 1). Lastly, subjects in the microglial analysis were: control group (male = 1, female = 2), RSG group (male = 1, female = 2), irradiated group (male = 1, female = 2), RSG-irradiated group (male = 2, female = 1).

### Immuno-Fluorescence and Stereology

Six sagittal brain sections spanning the dorsal hippocampus, (∼180 μm between sections) were collected for each animal using a Leica CM3050 S Cryostat (Leica Biosystems, Nubloch, Germany). Sections were submerged and washed in 1× PBS for 5 min, 3 times. Next, free-floating sections were permeabilized in 0.3% tween-1× PBS for 15 min and then blocked in 5% normal donkey serum in 1× PBS for 1 h at room temperature. After, samples were co-stained with rat anti-BrdU (1:1000; Abcam, ab6326) and either mouse anti-NeuN (1:750; Millipore, MAB377) for analysis of neuronal survival or rabbit anti-Ki67 (1:400; Cell Signaling, 9129s) to visualize cellular proliferation. Single probe studies included incubation with goat anti-doublecortin (1:250, SantaCruz, sc-8066) to measure immature neurons or with Iba-1 (1:200, WAKO, 019-19741) to study microglia populations. All samples were incubated with primary antibodies overnight at 4°C. After, sections were incubated with goat anti-doublecortin (Santa Cruz), mouse anti-NeuN (Millipore), rabbit anti-Ki67 (Cell signaling) and rabbit anti-Iba (WAKO) in 3%BSA-1×PBS, overnight at 4°C. Next, sections were washed 5 times in 1× PBS and next stained with Thermo Fisher Scientific Alexa-Fluor^®^ secondary antibodies (1:250): donkey anti-goat 488 nm (A-11055), donkey anti-rabbit 488 nm (A-21206) and 568 nm (A-10042) and donkey anti-mouse 647 nm (A-31571) for 1 h at room temperature in 3% BSA-1× PBS. Sections were mounted on SuperFrost^®^ glass slides and sealed in VectaSheild^®^ mounting media. Lastly brain sections were counterstained with 4′6-Diamidino-2-Phenlindole Dihydrochloride (1:5000; Millipore-Sigma, D9542) DAPI to visualize cellular nucleus. The hippocampus for all samples were captured using large image capture function on an A1 Nikon confocal microscope with a 40× objective water lens. Microglia coverage and roundness in the dentate gyrus were analyzed using Fiji’s (ImageJ) “Analyses of Particles” function. Briefly, threshold for 8-bit images was set for each section and Fiji’s “Magic Wand” was used to randomly select Iba-1 positive cells. A total of 90 positive cells for each group were measured i.e., five cells/section, six sections/animal and 3 animals/group. Samples were randomly chosen and experimenter was blinded to treatment groups upon quantitation of cellular markers.

### Data Analysis and Statistics

Data analysis and statistics were performed using GraphPad Prism software. Data was analyzed using univariate and multivariate ANOVAs for analysis of behavior area under the curve and immunofluorescence. Average area under the curve was calculated for each group by taking discrimination ratios for each day and setting baseline to zero. Analyses for cognitive performance was reported as mean % freezing ±SEM with 95% confidence intervals, where appropriate. Group interactions were tested using two-way ANOVA to compare RSG and irradiation effects in middle-aged B6SJL mice for immunofluorescence studies. The *F*-test for equal variance between groups was applied to all data sets. Context discrimination ratios were calculated and tested using a one sample *t*-test against chance, a theoretical mean of 0.5. Repeated measures two-way ANOVA using discrimination ratios was used to determine an effect of genotype vs. testing day. The Levene’s test was used to determine equal variances for within subject’s design (days). A within subject repeated measures two-way ANOVA using raw freezing data (total freezing Context A vs. total freezing Context B) was another measure for context discrimination. Statistical results are detailed in each figure legend.

## Results

### Neurobehavioral Analyses

**FIGURE 2:**
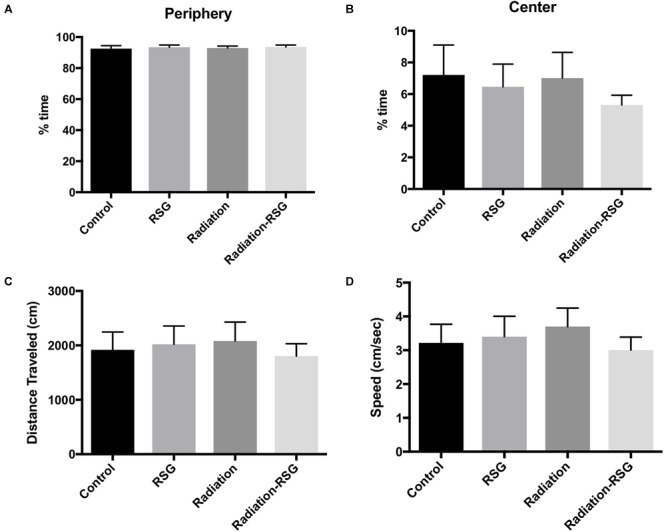
Radiation and rosiglitazone do not alter exploratory behavior in middle-aged mice. All subjects were allowed to explore an open field test box for 10 min to measure natural exploratory behavior in a novel context. **(A)** Two-way ANOVA (*F*_(1,59)_ = 0.00; *p* = 0.98) revealed control (mean = 93.01%) animals spent similar percent time exploring the periphery of the open field box as did groups treated with RSG (mean = 93.52%), radiation (mean = 92.92%) and RSG+Radiation (mean = 93.5). **(B)** Two-way ANOVA (*F*_(1,59)_ = 0.31; *p* = 0.58) test revealed controls (mean = 6.6%), RSG (mean = 6.4%), radiation (mean = 7.0%) and Radiation+RSG (mean = 5.2%) time exploring the center of the box was no different this study. **(C)** Average total distance traveled for controls (mean = 1859 cm), RSG (mean = 2081 cm), radiation (mean = 2080 cm) and Radiation+RSG (mean = 1828 cm) traveled similar distance throughout the trial; Two-way ANOVA (*F*_(1,59)_ = 0.63; *p* = 0.42). **(D)** Velocity for controls (mean = 3.04 cm/s), RSG (mean = 3.51 cm/s), radiation (mean = 3.7 cm/s) and RSG-Radiation (3.0 cm/s) were comparable in open field test; ANOVA (*F*_(1,59)_ = 1.33; *p* = 0.25).

To confirm that 30-day treatment of RSG or a single low dose cranial irradiation did not alter baseline behavior in mice, we tested natural exploration and anxiety like behavior by exposing all groups to a novel context in open field test for 10 min. Thirty days after initial treatments, percent time exploring the periphery and center of open field box was similar among groups (Figure 2A,B). Furthermore, there was no difference in total distance traveled or speed between groups (Figure 2C,D). Anxiety like behavior was equivalent between treatment and control groups. Thus, in all metrics of the open field test demonstrated that neither irradiation nor PPARγ agonist treatment, nor both, affected baseline behavior in this study.

**FIGURE 3:**
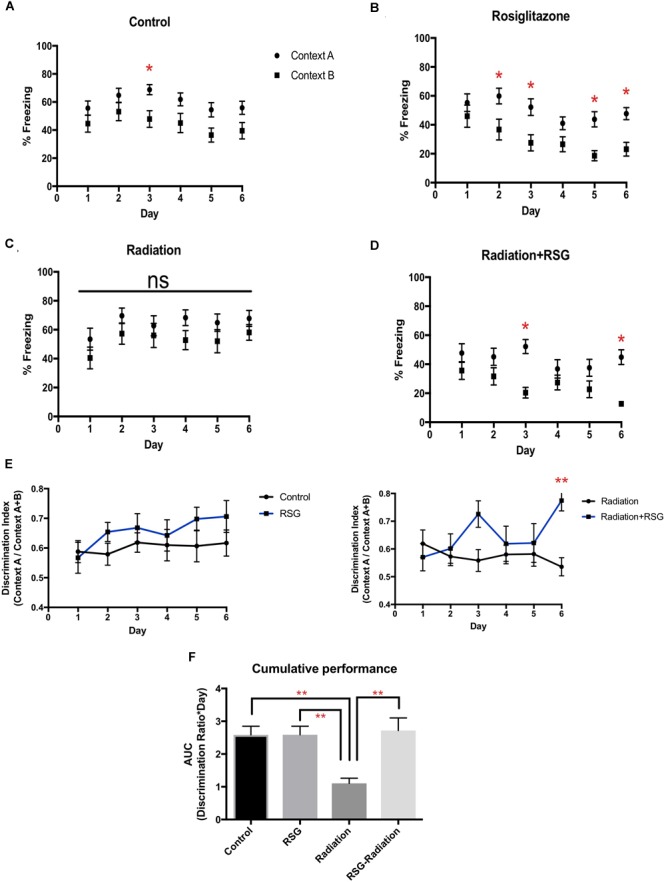
RSG treatment improved context discrimination in middle-aged and cranial irradiated mice. Percent time freezing in context A (circles) and context B (squares) for each treatment group. Data represented as mean ± SEM. ^∗^*p* < 0.05 **(A)** *Within* group two way ANOVA- repeated measures determined that middle-aged mice (*n* = 17), (Context vs. Day; *F*_(5,60)_ = 0.34, *p* = 0.88); Sidak’s *post hoc* pairwise comparisons determined significant discrimination on day 3. **(B)** RSG-treated mice (*n* = 17), (Context vs. Day) (*F*_(5,60)_ = 1.05, *p* = 0.38) discriminated on days 2, 3, 5, 6. **(C)** *Within* group two way repeated measures ANOVA and Sidak’s *post hoc* pairwise comparisons determined that discrimination was eliminated in cranial irradiated mice (*n* = 13) (Context vs. Day) (*F*_(5,60)_ = 0.127; *p* = 0.98), **(D)** while RSG-treated cranial irradiated mice (*n* = 17), (Context vs. Day) (*F*_(5,60)_ = 2.21; *p* = 0.055) discriminated on days 3, and 6. **(E)** Discrimination ratios were calculated by dividing total percent freezing in context A by percent freezing in both context A and B. RSG-treated groups showed improved learning by day 6 as measured with the discrimination index. Repeated measures two-way ANOVA *between groups* (*F*_(5,60)_ = 1.00, *p* = 0.44); Sidak’s *post hoc* determined significant discrimination on day 6 ^∗^Radiation vs. Radiation+RSG. **(F)** AUC calculations determined that RSG restored context discrimination in cranial irradiated animals. Two-way ANOVA (*F*_(1,20)_ = 8.4, ^∗∗^*p* < 0.01).

Cranial irradiation is an effective strategy to eradicate adult neurogenesis in preclinical models (Denny et al., 2012). Adult hippocampal neurogenesis in particular correlates with performance in pattern separation/context discrimination learning and memory tasks (Aimone et al., 2010; Sahay et al., 2011b). Therefore, to determine if PPARγ agonism influences context discrimination via a neurogenesis-dependent manner with and without focal cranial irradiation, we tested our groups in a context fear discrimination memory task (Figure 3). On day 2, middle-aged RSG-treated mice discriminated Context A from B. By day 3, control, RSG treated and irradiation+RSG groups froze significantly more in Context A than in similar safe Context B. However, irradiation treated group were not able to discriminate between shock and safe context over the entire course of this study indicating irradiation completely disrupts context discrimination. Thus, daily assessment of %freezing in Context A vs. Context B indicates that middle-aged animals’ performance is enhanced with RSG treatment and focal cranial irradiation obliterates context discrimination in middle-aged mice.

An additional metric for overall performance in context fear discrimination was calculated using area under the curve (AUC) and daily discrimination ratio calculation. With these analyses, we confirmed that PPARγ agonism restored discrimination performance diminished by irradiation exposure.

### Immunofluorescence

#### SGZ Cell Proliferation

**FIGURE 4:**
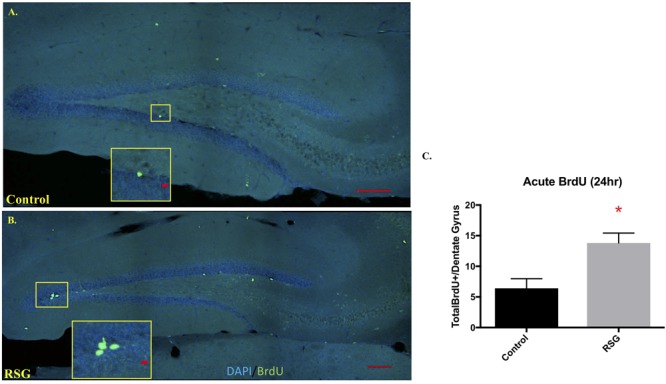
PPARγ agonism with RSG stimulated progenitor proliferation in hippocampus subgranular zone (SGZ). **(A)** Confocal images using sagittal sections of dorsal hippocampus from 9.5-months old mice treated with RSG (*n* = 5) or untreated (*n* = 5) following BrdU injection 24 h prior to trace dividing cells. DAPI (Blue) and BrdU (Green) show that recently divided cells are more abundant in subjects treated with RSG. **(B)** Dividing cells were significantly more abundant in dorsal hippocampi of RSG-treated mice (13.8, ± 1.62) compared to untreated 9.5 MO mice (6.4, ± 1.56). **(C)** Data represented as mean ± SEM. (^∗^*p* < 0.05), Student’s *t*-test).

**FIGURE 5:**
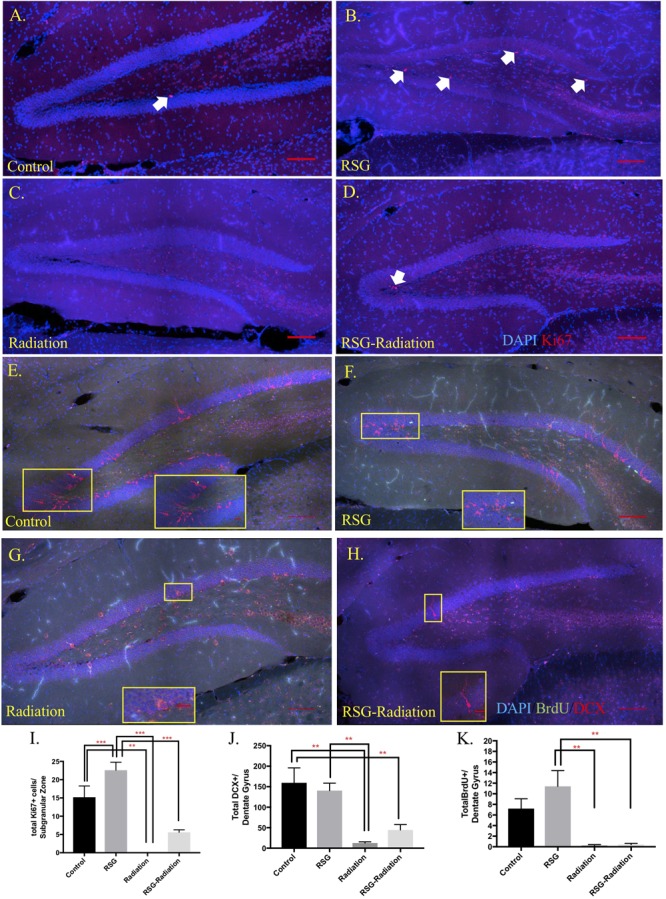
RSG stimulates neural progenitor cells but not immature neurons and newborn neuron survival in dorsal hippocampus. **(A–D)** Proliferating cells in the dorsal hippocampal SGZ were labeled with Ki67 (red). Ki67 positive cells were significantly increased in animals treated with RSG for 45 days. Irradiation and RSG had a significant effect on Ki67 positive cell. **(E–H)** Young neurons were labeled with the new neuron marker, doublecortin (DCX, red). Dorsal hippocampus section from control, RSG, irradiated, and RSG+irradiated, 9.5-month old mice. Long-term neuron survival in the granule cell layer of the dentate gyrus was unchanged with RSG treatment. Neuron survival as indicated with BrdU (green) positive cells (5-bromo-2’deoxyuridine-50 mg/kg) were significantly reduced in irradiated groups; **(I)** Interaction (*F*_(1,16)_ = 5.3, *p* = 0.03). Sidak’s *post hoc* analysis determined RSG significantly increased Ki67 immunostaining. Radiation greatly reduced immunostaining compared to control and RSG control groups. Two-way ANOVA ^∗∗^*p* < 0.01. **(J)** Abundance of doublecortin-positive neurons (DCX, red; DAPI, blue) in the dentate gyrus was similar between control and the RSG control group. Doublecortin labeling was greatly reduced following irradiation 45 days prior; Radiation (*F*_(1,16)_ = 26.3, ^∗∗^*p* < 0.001). **(K)** Two-way ANOVA Radiation (*F*_(1,16)_ = 26.13, ^∗∗∗^*p* < 0.001) (*n* = 5 mice/group).

Previous work reported that PPARγ agonism improved cell proliferation in subregions of brain ventricles and *in vitro* but has yet to be tested in mouse hippocampus (Morales-Garcia et al., 2011). Here, we show that RSG significantly increases neuronal precursor/proliferation marker in the subgranular zone (SGZ) of the hippocampus compared to untreated subjects (Figure 4). This result was confirmed by injecting middle-aged mice with mitotic marker 5-bromo-2-deoxyuridine (BrdU 100 mg/kg) and harvesting tissue 24 h later. Immunostaining for BrdU 24 h post-injection revealed increased proliferation in the SGZ of RSG treated mice compared to untreated, indicating PPARγ agonism stimulates self-renewing cells in the SGZ of the dorsal hippocampus. We confirmed that sub-granular zone proliferation was stimulated with RSG treatment by staining proliferating cells with Ki67. Quantification of Ki67 positive cells in the SGZ revealed a significant increase in proliferative cells which was obliterated with irradiation (Figure 5).

#### SGZ Neuron Differentiation

PPARγ agonism influences neurite growth *in vitro* and hippocampal immature neuron abundance (Ormerod et al., 2013) however, we show that the marker for immature neurons, doublecortin immunoreactive cells were not significantly different among groups (Figure 5). Furthermore, to determine if RSG affects newborn neuron survival, we treated 8-month old with RSG, as previously described. Twenty-four hours after irradiation treatment, mice were IP injected with 50 mg/kg/day of BrdU, for 5 consecutive days. Quantification of BrdU positive cells in the granule cell layer of the dorsal hippocampus revealed no effect of PPARγ agonism (Figure 5).

#### Neuroinflammation

**FIGURE 6:**
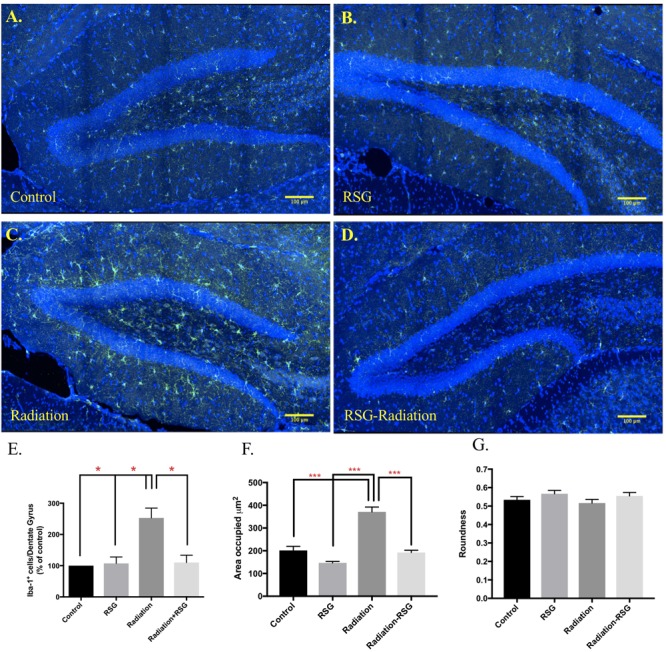
RSG treatment attenuated cranial irradiation-induced microglia response. Dorsal hippocampal sections from 9.5 months old mice left control **(A)**, and RSG-treated **(B)**, labeled for Iba-1 (green) and DAPI (blue) were captured using confocal microscopy. **(C)** The elevated levels of Iba-1-positive cells in the radiation group were significantly reduced with 45 days of RSG treatment, **(D)**. **(E)** Significance was established when (*F*_(3,8)_ = 0.00, ^∗^*p* < 0.05) compared to control group, two-way ANOVA followed by Sidak’s *post hoc* multiple comparisons test (*n* = 3/group). **(F,G)** Significantly larger area occupied by Iba-1 positive cells in irradiated group (*F*_(1,356)_ = 29.4), *p* < 0.0001; Sidaks *post hoc* ^∗∗∗^*p* < 0.0001. Roundness of selected cells were similar across all groups; (*F*_(1,356)_ = 0.02, *p* = 0.86).

Iba-1 positive (Iba-1^+^) microglia is an indicator of microglia that are responding to a CNS insult. Iba-1^+^ microglia perform complex processes including synaptic pruning, phagocytosis and inflammatory signaling (Li and Barres, 2018). Further, chronic microglia activation has been shown to be detrimental to hippocampal-dependent memory (Acharya et al., 2016). Here we show PPARγ agonism attenuated Iba-1^+^ microglia abundance in the dentate gyrus induced by cranial irradiation 45 days after initial treatment. Furthermore, RSG treatment restored abundance and area of microglia but roundness was unaltered with PPARγ agonism (Figure 6).

## Discussion

In this study, we found that PPARγ agonism enhanced context discrimination performance in middle-aged mice concomitant with stimulated SGZ cell proliferation (24 h BrdU incorporation and Ki67 staining), but not new neuron survival (45 dpi BrdU incorporation, doublecortin staining). Focal cranial irradiation that destroys neurogenesis, severely compromised context discrimination in middle-aged mice, yet rosiglitazone treatment significantly improved performance in the context discrimination fear conditioning task. In contrast to rosiglitazone effect on context discrimination in middle-aged mice, this improvement following focal cranial irradiation was accompanied by mild neuron production (doublecortin staining) and attenuated microglial response (Iba-1 staining), presumably due to chronic neuroinflammation resulting from focal cranial irradiation. We interpret these findings as indicative of divergent mechanisms for PPARγ agonism for aging-related vs. cranial irradiation-induced deficits in context discrimination.

In the present study, we demonstrated that PPARγ agonism restored context discrimination learning and memory following cranial irradiation concomitant with reduced hippocampal Iba-1+ reactive microglia as a signature for inflammation. Cranial radiotherapy for brain tumors, head and neck cancer etc., in humans triggers chronic neuro-inflammation and interferes with adult neurogenesis that leads to memory impairment (Liyuan et al., 2015) that can last months to years after treatment is complete (Ramanan et al., 2010). Therefore, therapeutic strategies that ameliorate adverse effects of cranial radiotherapy should be considered. Since the effects of RSG following focal cranial irradiation attenuated neuroinflammation induced by cranial irradiation treatment.

Using exploration in an open field chamber, we determined that baseline activity was not altered by radiation exposure as all groups explored center and peripheral arenas equally as well as locomoting equivalent total distance. Furthermore, freezing in context A on day 0 prior to the foot shock and in completely novel context C on day 1 were comparable among groups, confirming that natural exploration behavior was unaltered by irradiation and RSG treatments, in this study (data not shown). Conditioned fear, as measured by freezing, was comparable in the first few days of testing suggesting all groups in both cohorts were able to recall the foot shock in context A. It appears that RSG-irradiation and RSG only subjects learn context B is not aversive as freezing declined over the course of the study, while freezing in context A remained consistent. Thus, PPARγ agonism promotes the ability to distinguish highly similar contexts with persistent training.

We also assessed learning by calculating discrimination ratios on a daily basis. That is, calculating the proportion of freezing in context A as a function of total freezing in contexts A and B. Based on discrimination ratios, RSG ameliorated context discrimination impairment induced by focal cranial irradiation or aging. Again, PPARγ agonism did not alter initial learning or memory, as measured with discrimination ratios, but with repeated training, RSG treatment improved performance compared to untreated groups. Area under the curve analysis, a third way to analyze context discrimination performance, also demonstrated that PPARγ agonism improved context discrimination compared to untreated groups.

Multiple studies demonstrated that adult hippocampal neurogenesis, in particular within the dorsal hippocampus, correlates with performance in context discrimination learning and memory (Frankland et al., 1998; Sahay et al., 2011a,b). As well, exercise and genetic manipulations that promote proliferation and maturation of adult born hippocampal neurons have positive effects on context discrimination learning and memory (Creer et al., 2010; Sahay et al., 2011a; Wu et al., 2015). The literature on the effect of PPARγ agonism on neuronal proliferation and differentiation is somewhat murky in that some claim it increased whereas another reported that it decreased hippocampal neurogenesis in young adult rodents (Quintanilla et al., 2014). We therefore set out to determine if RSG influenced either proliferation within the SGZ or survival of adult born neurons.

Since focal cranial irradiation obliterated BrdU immunore- active cells in the dentate gyrus 45 dpi, we instead compared control and RSG-treated groups to determine the effect of PPARγ agonism on neurogenesis markers. We found that 45 days of RSG improved proliferation (BrdU incorporation 24 h after injection), but not differentiation and survival of adult born neurons in the dorsal hippocampus of 9.5-month old mice (doublecortin and BrdU staining 45 dpi). However, after a single dose of 10 Gy cranial irradiation, 45 days of PPARγ agonism moderately boosted doublecortin expressing neuroblast-like cells in the SGZ, indicating that PPARγ agonism positively influences the neurogenic niche. However, what we don’t know whether these doublecortin positive SGZ cells are contributing to new neuron incorporation in the dentate gyrus to affect context discrimination learning and memory. These results indicated that PPARγ agonism stimulated hippocampal SGZ proliferation but not neuronal maturation and survival in middle-aged mice. Although the histogram for increased adult born neuron survival (BrdU-staining) suggests enhancement with 45 days RSG treatment, the effect was not significant and we attribute the rather large error bar to one of five RSG-treated samples showing a large deviation upward in positive cell counts. Thus, while PPARγ agonism acutely stimulates SGZ cell proliferation, it does not affect neuron survival.

Microglial proliferation following cranial irradiation has also been linked to memory impairment and chronic neuroinflammation (Acharya et al., 2016). PPARγ agonism with RSG has been shown to modify a repertoire of inflammatory markers (Shiojiri et al., 2002), therefore we evaluated microglia in the hippocampi of the focal cranial irradiation cohort. Microglia are resident immune cells in the brain that are necessary to maintain homeostasis following insults such as cranial irradiation (Li and Barres, 2018). While microglia perform complex neuro-immune processes, like synaptic pruning and cytokine/chemokine production, microglia can become toxic in a feed forward manner during chronic infections and neurodegenerative disease. Using Iba-1 as a marker for microglia, we found that RSG reversed the area occupied and the abundance of microglia staining resulting from focal cranial irradiation. This suggested to us that improved context discrimination in RSG-irradiated subjects was due to amelioration of chronic neuroinflammation as measured by numbers of microglia rather than effects on neurogenesis as indicated by the presence of doublecortin positive neuroblasts in the SGZ of the dentate gyrus 45 days post-cranial irradiation.

In middle-aged mice, PPARγ agonism with RSG improved performance without effect on adult born neuron production or survival as measured with doublecortin and BrdU. These results are similar to findings from Wu et al. (2015), where improved context discrimination was observed without evidence of increased adult hippocampal neurogenesis in aged mice subjected to *ad libitum* wheel running. Following focal cranial irradiation, RSG treatment rescued context discrimination learning and memory via an anti-inflammatory mechanism (amelioration of Iba-1-positive microglia) and resurrection of the neurogenic niche. Altogether, our results show that PPARγ agonism supports context discrimination memory in middle-aged mice and mice exposed to cranial irradiation via distinct mechanisms. Thus, RSG treatment might be a putative therapeutic for conditions accompanied by neuroinflammation such as Alzheimer’s disease, multiple sclerosis or radiotherapy for brain cancer. Finally, PPARγ agonism as a prophylactic may provide cognitive benefits during aging.

## Author Contributions

IC and KD analyzed the data and wrote the manuscript. IC performed all the experiments. All authors designed the research and discussed the findings during manuscript preparation.

## Conflict of Interest Statement

The authors declare that the research was conducted in the absence of any commercial or financial relationships that could be construed as a potential conflict of interest.
